# *Lycium barbarum* Polysaccharides as Antibiotic Substitutes Improve Growth Performance, Serum Immunity, Antioxidant Status, and Intestinal Health for Weaned Piglets

**DOI:** 10.3389/fmicb.2021.819993

**Published:** 2022-02-25

**Authors:** Yexin Yin, Fang Wang, Mei Yang, Bie Tan, Yulong Yin, Jiashun Chen, Zhe Yang

**Affiliations:** ^1^Animal Nutritional Genome and Germplasm Innovation Research Center, College of Animal Science and Technology, Hunan Agricultural University, Changsha, China; ^2^Key Laboratory of Agro-ecological Processes in Subtropical Region, Scientific Observing and Experimental Station of Animal Nutrition and Feed Science in South-Central, Ministry of Agriculture, Hunan Provincial Engineering Research Center of Healthy Livestock, Institute of Subtropical Agriculture, Chinese Academy of Sciences, Changsha, China

**Keywords:** antioxidant, growth performance, immune, intestinal health, *Lycium barbarum* polysaccharides, weaned piglets

## Abstract

The aim of the present study is to investigate the effects of dietary *Lycium barbarum* polysaccharides (LBPs) supplementation on the growth performance, immune response, serum antioxidant status, and intestinal health of weaned piglets. In total, 24 crossed healthy weaned piglets [Duroc × (Yorkshire × Landrace)], of similar body weight (7.47 ± 0.22 kg), were randomly allocated to three treatment groups: CON (basal diet); LBPs (basal diet plus 4,000 mg/kg LBPs); and antibiotic (ABO, basal diet plus 20 mg/kg flavomycin and 50 mg/kg quinocetone). There were eight pigs per group. The study lasted 28 days. When compared with CON, LBPs or ABO dietary supplementation increased average daily gain (*P* < 0.05), decreased the ratio of feed to gain and the diarrhea ratio (*P* < 0.05). Similarly, when compared with CON, LBPs dietary supplementation increased serum immunoglobulin G, immunoglobulin M, interleukin-10, interleukin-2, and tumor necrosis factor-α levels (*P* < 0.05). Dietary LBPs enhanced the activity of serum total antioxidant capacity and glutathione peroxidase, and decreased malondialdehyde levels (*P* < 0.05). Principal component analysis showed a distinct separation between CON and LBPs groups, but no differences between ABO and LBPs groups. LBPs addition increased *Lactobacillus* and *Faecalibacterium* (*P* < 0.05) levels, while it decreased *Enterococcaceae* and *Enterobacteriaceae* (*P* < 0.05) levels. Furthermore, when compared with the CON group, LBPs increased villus height (*P* < 0.05) and the villus height to crypt depth ratio in the duodenum and jejunum (*P* < 0.05). Thus, dietary supplementation with LBPs improved growth performance, antioxidant capacity and immunity, regulated intestinal microbial composition, and may be used as an efficient antibiotic alternative in weaned piglet feed.

## Introduction

Early weaning increases intestinal permeability and reduces antioxidant capacity and immunity, which reduces feed intake, and increases diarrhea incidence, morbidity, and mortality (Hu et al., 2013; Yin et al., 2014). Diarrhea after weaning is mainly associated with gut microbiome disturbances which may lead to fever and slow growth (Campbell et al., 2013). Antibiotics are widely used in animal feeds to regulate intestinal microorganisms, prevent infection, and improve growth performance (Cook, 2004; Wang W. et al., 2018). However, antibiotics over-dependence has facilitated the emergence of antimicrobial resistance and antimicrobial residues, which affect human health (Li, 2017). In the European Union, antibiotics in feed additives were banned in 2006, whereas, in China, their use ceased in July 2020, therefore, a healthy and pollution-free alternative to antibiotics is required.

Many plant extracts can be used as alternatives to antibiotics (Lu et al., 2010; Pourhossein et al., 2015). *Lycium barbarum*, as a food and medicine, has been used in Asian countries for thousands of years to induce various health benefits (Donno et al., 2015; Zhao J. et al., 2016). *L. barbarum* polysaccharides (LBPs) are major bioactive components of *L. barbarum* and possess distinct bioactivities, including anti-oxidant (Wang et al., 2020; Zhang et al., 2021), anti-tumor (Gong et al., 2020), anti-diabetic (Shimato et al., 2020), immunomodulatory (Feng et al., 2020; Kim et al., 2020), liver protective (Jia et al., 2016), neuroprotective (Zhao Z. et al., 2016), renal protective (Wu et al., 2020), and improved eyesight activities (Zhu et al., 2016). Liu et al. (2021a) demonstrated that variations in the molecular weight of LBPs exerted antioxidant effects on different free radical. Yang et al. (2013) indicated that LBPs treatment may protect intestinal damage by inhibiting oxidative stress and inflammation in rats. Long et al. (2020) reported that dietary supplementation of LBPs could improve the growth performance, immune function, antioxidant capacity, and digestive enzyme activities in broilers. Our previous studies demonstrated that 4,000 mg/kg LBPs dietary supplementation enhanced growth performance, immune status and antioxidant capacity, and improved intestinal microbial populations in weaned piglets (Chen et al., 2020). Based on these favorable effects, we hypothesized that dietary LBPs supplementation could effectively replace antibiotics by improving performance, gastrointestinal tract health, and function in weaned piglets. Therefore, the objective of the current study was to investigate the effects of a 4,000 mg/kg LBPs supplementation on growth performance, diarrhea incidence, serum immunity and antioxidant capacity, intestinal morphology, short-chain fatty acids (SCFAs) levels, and cecum intestinal microflora in weaned pigs.

## Materials and Methods

Experiments were conducted in accordance with Chinese guidelines for animal welfare and experimental protocols. All animal procedures were approved by the Committee of Animal Care at Hunan Agricultural University (Changsha, China) (permit number: CACAHU 2020-00156).

### Experimental Design

We included 24 crossed healthy weaned piglets [Duroc × (Yorkshire × Landrace)] of similar body weight (BW = 7.47 ± 0.22 kg). Animals were randomly allocated to three treatment groups: CON (basal diet); LBPs (basal diet plus 4,000 mg/kg LBPs); and antibiotic (ABO, basal diet plus 20 mg/kg flavomycin & 50 mg/kg quinocetone). There were eight pigs per group. The basal diet was formulated to satisfy or outstrip National Research Council (National Research Council, 2012) nutrient requirements. Basal diet nutrient levels and ingredients are shown (Table 1).

**TABLE 1:** Ingredients and chemical composition of experimental diets (as-fed basis).

Items	Content (%)
**Ingredients**	
Corn	55.00
Soybean meal	19.00
Full-fat soybean powder	10.00
Fish meal	5.00
Whey powder	6.15
Soybean oil	1.50
Dicalcium phosphate	0.90
L-Lysine-HCl	0.48
L-Threonine	0.05
DL-Methionine	0.10
L-Tryptophan	0.02
Salt	0.30
Limestone	0.50
Premix^a^	1.00
Total	100.00
**Calculated nutrients**	
Digestible energy (MJ/kg)	14.64
Crude protein	20.15
Lysine	1.38
Methionine	0.82
Methionine + cysteine	1.01
Threonine	0.97
Tryptophan	0.25
Calcium	0.80
Total phosphorus	0.73

*^a^The premix provided the following (per kilogram of compound feed): Vitamin A, 12,000 IU; Vitamin D, 2,500 IU; Vitamin E, 30 IU; Vitamin B12, 12 μg; Vitamin K, 3 mg; d-pantothenic acid, 15 mg; nicotinic acid, 40 mg; choline chloride, 400 mg; Mn, 40 mg; Zn, 100 mg; Fe, 90 mg; Cu, 8.8 mg; I, 0.35 mg; Se, 0.3 mg.*

All pigs were housed in a room with slatted floors. They were fed in individual metabolism cages with a side feeder and a stainless-steel nipple which provided full access to feed and water, respectively. The scale of feeding and feed surplus for each piglet was recorded throughout the study. At study beginning and end, body weights were measured; these data were used to calculate the average daily gain (ADG), average daily feed intake (ADFI), and ratio of feed to gain (F/G). The study lasted for 28 days and diarrhea ratio was monitored daily. Diarrhea ratio (%) was calculated as the number of pigs with diarrhea × the number of days with diarrhea/(the total number of pigs × the number of study days) (Hung et al., 2019).

### Sample Collection and Preparation

On the 27th day, blood was collected by anterior vena cava puncture before morning feeding. Blood was centrifuged at 3,000 × *g* for 15 min at 4°C to isolate serum which was stored at –80°C. All piglets were humanely killed by injection of pentobarbital sodium at study end and the gut, liver, and kidney immediately removed from the abdominal cavity. The intestinal segment and mucosa from the duodenum, jejunum, and ileum were collected and stored at –80°C. An intestinal segment (comprising duodenum, jejunum, and ileum) was fixed in 4% paraformaldehyde-phosphate buffered saline buffer to analyze intestinal morphological structures. Chyme from the ileum, cecum, and colon was collected and stored at –80°C.

### Immune Responses

Serum immunoglobulins (Ig)A, IgM; IgG, the interleukins, (IL)-2, IL-6, IL-10, IL-1α, and IL-1β; and tumor necrosis factor-α (TNF-α) were measured by using pig-specific ELISA kits (Cusabio Biotechnology Co., Ltd., Wuhan, China).

### Antioxidant Capacity

The activity levels of total antioxidant capacity (T-AOC), superoxide dismutase (SOD), glutathione peroxidase (GSH-Px), and malondialdehyde (MDA) in serum were determined using respective reagent kits (Nanjing Jiancheng Bioengineering Institute, Nanjing, China).

### Intestinal Morphology

Sections of the duodenum, jejunum, and ileum in each pig were harvested and immediately fixed in 10% formalin, dehydrated in 50% ethanol, embedded paraffin, and sectioned 5 μm for hematoxylin and eosin staining. The sections were scanned using an optical binocular microscope connected to a digital camera (Nikon ECLIPSE 80i). Villus length, crypt depth, and the villus length vs. crypt depth (V/C) ratios were measured from 10 well-oriented villi × 3 sections of each pigs.

### Gut Microbiota Analysis

According to the manufacturer’s instructions, total genomic DNA was extracted from the chyme of cecum samples using the QIAamp Fast DNA stool mini kit (Qiagen, Hilden, Germany). DNA was checked on 1% agarose gels and concentration and purity were determined using a NanoDrop 2000 UV–vis spectrophotometer (Thermo Fisher Scientific, Wilmington, United States). The V3–V4 hypervariable region of the bacterial 16S rRNA gene was amplified using the following primers; 338 F (5′- ACTCCTACGGGAGGCAGCAG-3′) and 806 R (5′-GGACTACHVGGGTWTCTAAT-3′) on an ABI GeneAmp^®^ 9700 PCR thermocycler (ABI, CA, United States) (Xu et al., 2016). The PCR amplification system and conditions have been previously described (Yang J. et al., 2020). PCR products were extracted from 2% agarose gel and purified using the AxyPrep DNA gel extraction kit (Axygen Biosciences, Union City, CA, United States) according to manufacturer’s instructions and quantified using a Quantus™ Fluorometer (Promega, United States).

Purified amplicons were pooled in equimolar quantities and paired-end sequenced on an Illumina MiSeq PE300 platform/NovaSeq PE250 platform (Illumina, San Diego, CA, United States) according to standard protocols of Majorbio Bio-Pharm Technology Co., Ltd. (Shanghai, China). Raw reads were deposited into the National Center for Biotechnology Information Sequence Read Archive database (Accession Number: SRP342805).

Raw 16S rRNA gene sequencing reads were demultiplexed, quality-filtered by fastp version 0.20.0 (Chen et al., 2018), and merged by FLASH version 1.2.7 (Magoc and Salzberg, 2011) using the following criteria: (1) the 300 base pair (bp) reads were truncated at any site receiving an average quality score < 20 over a 50 bp sliding window. Truncated reads < 50 bp and reads containing ambiguous characters were also discarded; (2) only overlapping sequences longer than 10 bp were assembled according to their overlapped sequences. The maximum mismatch ratio of the overlap region was 0.2. Reads that could not be assembled were discarded; and (3) samples were distinguished according to the barcode and primers, and the sequence direction was adjusted, exact barcode matching, 2 nucleotide mismatch in primer matching.

Species diversity was evaluated using ACE and Chao richness estimators and Shannon and Simpson diversity indices (Lemieux-labonte et al., 2017). Operational taxonomic units (OTUs), with 97% similarity cutoff (Stackebrandt and Goebel, 1994; Edgar, 2013), were clustered using UPARSE version 7.1 (Edgar, 2013), with chimeric sequences identified and removed. Beta diversity was evaluated using Principal Component Analysis (PCA). Significant differences between samples were evaluated by analysis of similarities (ANOSIM).

### Determination of Intestinal Short-Chain Fatty Acid Levels

We performed gas chromatography (GC) to determine the main SCFAs in intestinal chyme, as described previously (Franklin et al., 2002). Briefly, to isolate supernatants, digesta samples were weighed, vortexed in distilled water, and centrifuged at 12,000 × *g* for 15 min at 4°C. Supernatants were mixed with 25% metaphosphoric acid at a 9:1 volume ratio, statically reacted for 3–4 h, centrifuged, and filtered. A GC system (GC2014, Shimadzu Corporation, Kyoto, Japan) was used to measure filtered fluids.

### Statistical Analysis

Experimental data were analyzed by one-way ANOVA using the General Linear Model procedure of the SPSS software v. 20.0 (SPSS Inc., Chicago, IL, United States). Differences between treatment means were tested using Tukey’s multiple comparison test. Microbe abundance, with significant differences between groups, was assessed by the Kruskal–Wallis test. Results were presented as the mean ± standard error of the mean. *P* < 0.05 was considered statistically significant.

## Results

### Growth Performance and Diarrhea Incidence

As shown in Table 2, when compared with the CON group, both LBPs and ABO dietary supplementation significantly increased ADG (*P* < 0.05) and decreased the F/G (*P* < 0.05). However, neither dietary LBPs or ABO supplementation had significant effects on initial weight, final weight, or ADFI in weaned piglets (*P* > 0.05).

**TABLE 2:** Effects of dietary LBPs supplementation on growth performance of weaned piglets.

Items^1^	Treatments^2^			SEM^3^	*P*-value
	CON	ABO	LBPs		
Initial weight, kg	7.47	7.48	7.47	0.204	1.000
Final weight, kg	16.5	17.3	17.6	0.297	0.317
ADG, g	323^b^	351^a^	362^a^	4.38	0.004
ADFI, g	563	584	572	7.28	0.492
F/G	1.75^a^	1.66^b^	1.58^b^	0.006	<0.001

*^1^ADFI, average daily feed intake; ADG, average daily gain; F/G, Feed/gain.*

*^2^Treatments consisted of (1) CON; basal diet, (2) LBPs; basal diet + 4,000 mg/kg LBPs and (3) ABO; basal diet + 20 mg/kg flavomycin + 50 mg/kg quinocetone.*

*^3^SEM, pooled standard error of mean (n = 8).*

*^a,b^Means within each row with different superscripts differ significantly (P < 0.05).*

**FIGURE 1:**
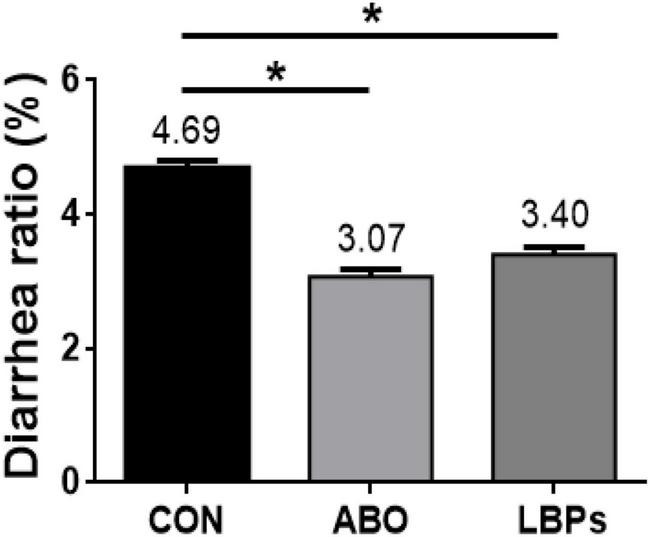
Diarrhea rate of weaned piglets fed MB dietary treatments (%) (*n* = 8). CON, basal diet; LBPs, basal diet + 4,000 mg/kg LBPs; ABO, basal diet + 20 mg/kg flavomycin + 50 mg/kg quinocetone. Asterisks express statistical differences between different groups: *P < 0.05.

As shown (Figure 1), when compared with the CON group, both LBPs and ABO dietary supplementation decreased diarrhea ratios in weaned piglets (*P* < 0.05), but no significant differences were observed between the LBPs and ABO groups (*P* > 0.05).

### Serum Immune Indices

As shown in Table 3, weaned piglets in the LBPs and ABO groups displayed higher IgG and IgM levels than the CON group (*P* < 0.05), but no significant differences were observed for IgA levels among the groups (*P* > 0.05). When compared with the CON group, LBPs dietary supplementation significantly increased serum IL-10, IL-2, and TNF-α (*P* < 0.05) levels, but no significant IL-6, IL-1α, and IL-1β differences were observed between the groups (*P* > 0.05) (Table 4).

**TABLE 3:** Effects of dietary LBPs supplementation on immune response in serum of weaned piglets.

Items^1^	Treatments^2^			SEM^3^	*P*-value
	CON	ABO	LBPs		
IgA, g/L	2.73	2.88	2.68	0.056	0.386
IgG, g/L	8.98^b^	11.2^a^	10.6^a^	0.302	0.018
IgM, g/L	0.09^b^	0.12^a^	0.11^a^	0.003	0.013

*^1^IgA, Immunoglobulin A; IgG, Immunoglobulin G; IgM,: Immunoglobulin M.*

*^2^Treatments consisted of (1) CON; basal diet, (2) LBPs; basal diet + 4,000 mg/kg LBPs and (3) ABO; basal diet + 20 mg/kg flavomycin + 50 mg/kg quinocetone.*

*^3^SEM, pooled standard error of mean (n = 8).*

*^a,b^Means within each row with different superscripts differ significantly (P < 0.05).*

**TABLE 4:** Effects of dietary LBPs supplementation on immunologic factors levels in serum of weaned piglets.

Items^1^	Treatments^2^			SEM^3^	*P*-value
	CON	ABO	LBPs		
IL-2, pg/ml	90.9^b^	98.4^b^	112^a^	2.53	0.010
IL-6, pg/ml	6.36	5.15	5.39	0.274	0.187
IL-10, pg/ml	11.7^b^	13.8^a^	13.5^a^	0.317	0.036
IL-1α, pg/ml	255	263	256	6.57	0.857
IL-1β, pg/ml	24.5	23.4	21.8	0.953	0.512
TNF-α, pg/ml	0.29^b^	0.30^b^	0.35^a^	0.006	0.004

*^1^IL, Interleukin; TNF-α, Tumor necrosis factor-α.*

*^2^Treatments consisted of (1) CON; basal diet, (2) LBPs; basal diet + 4,000 mg/kg LBPs and (3) ABO; basal diet + 20 mg/kg flavomycin + 50 mg/kg quinocetone.*

*^3^SEM, pooled standard error of mean (n = 8).*

*^a,b^Means within each row with different superscripts differ significantly (P < 0.05).*

### Antioxidant Capacity

Table 5 presents the differences in serum antioxidant indicators between the treatment groups. Dietary LBPs effectively enhanced serum T-AOC and GSH-Px activities but decreased MDA levels (*P* < 0.05). No significant differences in SOD activities were observed between the groups (*P* > 0.05).

**TABLE 5:** Effects of dietary LBPs supplementation on serum antioxidant activity of weaned piglets.

Items^1^	Treatments^2^			SEM^3^	*P*-value
	CON	ABO	LBPs		
T-AOC, U/mL	2.91^b^	3.31^a^	3.24^a^	0.045	0.004
GSH-Px, U/mL	319^b^	347^a^	338^a^	2.49	0.001
SOD, U/mL	189	184	197	3.81	0.403
MDA, nmol/mL	7.08^a^	4.81^b^	5.41^b^	0.224	0.001

*^1^T-AOC, Total antioxidant capacity; GSH-Px, Glutathione peroxidase; SOD, Superoxide dismutase; MDA, Malondialdehyde.*

*^2^Treatments consisted of (1) CON; basal diet, (2) LBPs; basal diet + 4,000 mg/kg LBPs and (3) ABO; basal diet + 20 mg/kg flavomycin + 50 mg/kg quinocetone.*

*^3^SEM, pooled standard error of mean (n = 8).*

*^a,b^Means within each row with different superscripts differ significantly (P < 0.05).*

### Intestinal Morphology

The effects of LBPs dietary supplementation on intestinal morphology in piglets at day 28 are shown in Table 6. When compared with the CON group, LBPs increased villus height in the duodenum and ileum (*P* < 0.05). A distinct decrease in crypt depth in the duodenum of piglets fed ABO was observed when compared with the CON group (*P* < 0.05). In addition, both LBPs and ABO dietary supplementation increased the V/C in the duodenum and jejunum when compared with the CON group (*P* < 0.05).

**TABLE 6:** Effects of dietary LBPs supplementation on intestinal morphology of weaned piglets.

Items^1^	Treatments^2^			SEM^3^	*P*-value
	CON	ABO	LBPs		
**Villus height, μm**					
Duodenum	289^b^	336^a^	362^a^	6.649	0.001
Jejunum	286	304	327	8.925	0.198
Ileum	234^b^	242^b^	289^a^	7.768	0.017
**Crypt depth, μm**					
Duodenum	268^a^	224^b^	264^a^	5.448	0.005
Jejunum	207	169	177	7.714	0.138
Ileum	149	150	142	5.361	0.807
**V/C, μm: μm**					
Duodenum	1.08^b^	1.52^a^	1.38^a^	0.034	<0.001
Jejunum	1.41^b^	1.83^a^	1.92^a^	0069	0.014
Ileum	1.60	2.08	1.69	0.086	0.076

*^1^V/C, the Villus height to Crypt depth rate.*

*^2^Treatments consisted of (1) Control; basal diet, (2) LBPs; basal diet + 4,000 mg/kg LBPs and (3) ABO; basal diet + 20 mg/kg flavomycin + 50 mg/kg quinones.*

*^3^SEM, pooled standard error of mean (n = 8).*

*^a,b^Means within each row with different superscripts differ significantly (P < 0.05).*

### Intestinal Microflora

**FIGURE 2:**
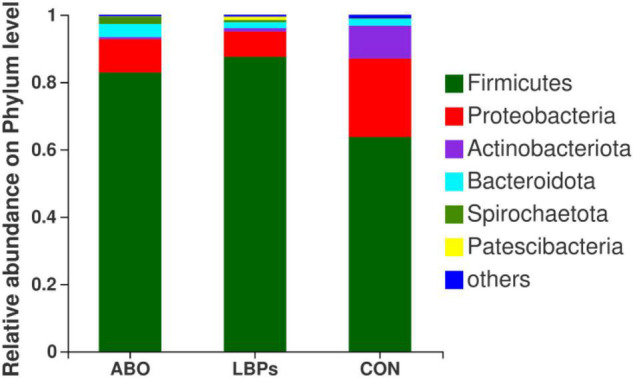
Phylum-level relative abundance of 16S rRNA gene sequences from the cecal digesta of weaned piglets (*n* = 8). CON, basal diet; LBPs, basal diet + 4,000 mg/kg LBPs; ABO, basal diet + 20 mg/kg flavomycin + 50 mg/kg quinocetone.

**FIGURE 3:**
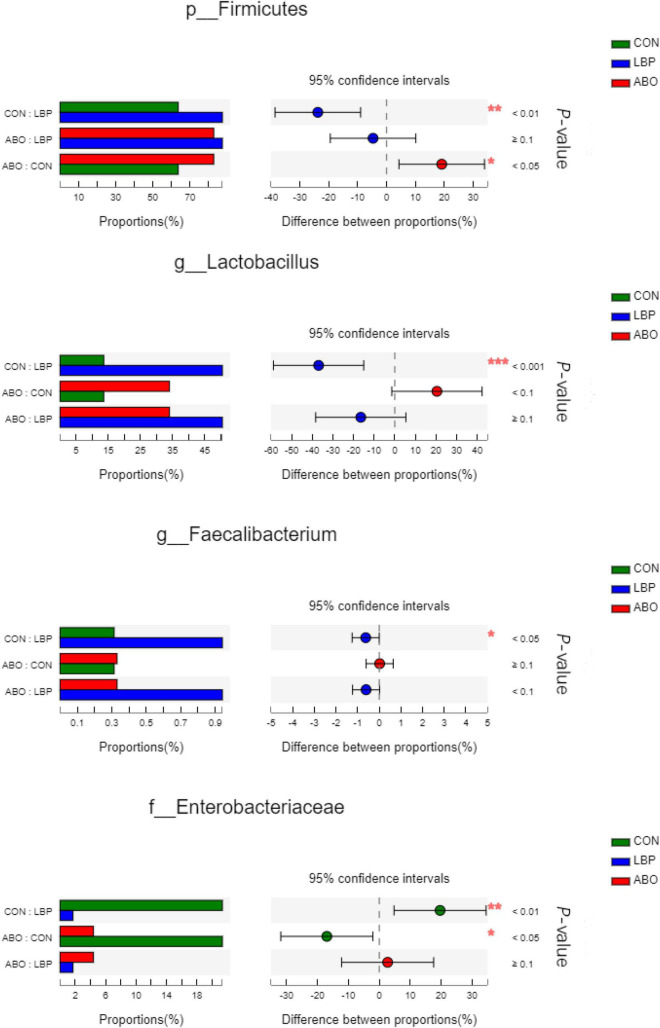
Comparative analysis of 3 most relative abundances of gut microbiota (*n* = 8). Kruskal–Wallis test followed by Tukey test was used to evaluate the statistical significance. Asterisks express statistical differences between different groups: *0.01 < *P* ≤ 0.05, **0.001 < *P* ≤ 0.01, ***P ≤ 0.001. CON, basal diet; LBP, basal diet + 4,000 mg/kg LBPs; ABO, basal diet + 20 mg/kg flavomycin + 50 mg/kg quinocetone.

**FIGURE 4:**
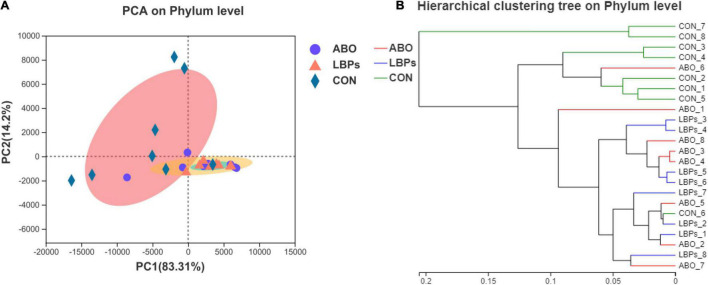
**(A)** Principal Component Analysis (PCA) of bacterial communities in the cecal digesta of weaned piglets (based on the Bray–Curtis distance) (*n* = 8). **(B)** Analysis of hierarchical clustering tree on Phylun level showed that the microbial composition of CON was almost entirely gathered in another branch. CON, basal diet; LBPs, basal diet + 4,000 mg/kg LBPs; ABO, basal diet + 20 mg/kg flavomycin + 50 mg/kg quinocetone.

In total, 1,216,334 high-quality sequences were obtained from samples. After clustering at the 97% similarity level, sequences were assigned to 905 OTUs. Firmicutes were the most abundant phylum across all samples, followed by Proteobacteria, Actinobacteriota, Bacteroidota, Spirochaetota, Desulfobacterota, and Patescibacteria (Figure 2). When compared with the CON group, the relative abundance of Firmicutes was significantly increased (*P* < 0.05) in the ABO and LBPs groups (Figure 3). Alpha diversity analyses indicated that LBPs increased Chao and ACE indices when compared with the CON group (*P* < 0.05), but no significant differences were observed for Shannon and Simpson indices among the groups (Supplementary Figure 1). PCA showed a distinct separation between the CON and LBPs groups, but no differences between the ABO and LBPs groups (Figure 4A). Hierarchical clustering tree analyses showed that CON microbial composition had mostly gathered in another branch (Figure 4B). From ANOSIM analyses, significant differences were identified in the microbial composition of the study groups; *r* = 0.2702, *P* < 0.01 in the CON, LBPs, and ABO groups; *r* = 0.2907, *P* < 0.05 for the ABO vs. CON groups; *r* = 0.4827, *P* < 0.01 for the LBPs vs. CON groups; and *r* = 0.0558, *P* = 0.185 for the ABO vs. LBPs groups). Additionally, *Lactobacillus* and *Faecalibacterium* were enriched (Figure 3) in the LBPs group at the genus level (*P* < 0.05), while *Enterobacteriaceae* (Figure 3), *Enterococcaceae*, and *Escherichia-Shigella* (Supplementary Figure 2) were enriched in the CON group (*P* < 0.05).

### Short-Chain Fatty Acids Levels

Total SCFAs, as well as acetic, propionic, isobutyric, butyric, isopentoic, and valeric acid levels in the cecum, ileum, and colon are shown in Table 7. When compared with the CON group, dietary both LBPs and ABO supplementation increased acetic, propionic and butyric acid levels, and total SCFAs, in the cecum (*P* < 0.05). However, no significant differences were observed in total ileum SCFAs or each SCFAs across groups (*P* > 0.05). Piglets fed the LBPs diet showed increased isobutyric and isopentoic acid levels in the colon when compared with the other groups (*P* < 0.05).

**TABLE 7:** Effects of dietary LBPs supplementation on short-chain fatty acids in intestinal contents of weaned piglets (μg/kg).

Items^1^	Treatments^2^			SEM^3^	*P*-value
	CON	ABO	LBPs		
**Cecum**					
Acetic acid	3.32^b^	6.00^a^	7.77^a^	0.336	<0.001
Propionic acid	2.84^b^	4.27^a^	4.30^a^	0.215	0.017
Isobutyric acid	0.871	0.771	0.719	0.006	0.549
Butyric acid	2.40^b^	3.75^ab^	4.57^a^	0.320	0.035
Isopentoic acid	0.149^a^	0.090^b^	0.083^b^	0.007	0.001
Valeric acid	0.606^a^	0.324^b^	0.471^ab^	0.038	0.022
Total SCFAs	9.40^b^	14.5^a^	17.3^a^	0.865	0.004
**Ileum**					
Acetic acid	0.546	0.623	0.647	0.044	0.622
Propionic acid	0.114	0.103	0.106	0.002	0.186
Isobutyric acid	0.019	0.017	0.016	0.001	0.609
Butyric acid	0.083	0.068	0.059	0.006	0.324
Isopentoic acid	0.007	0.010	0.007	0.001	0.398
Valeric acid	0.009	0.008	0.008	0.001	0.133
Total SCFAs	0.777	0.829	0.844	0.052	0.864
**Colon**					
Acetic acid	2.46	2.95	2.75	0.115	0.246
Propionic acid	1.89	1.50	1.12	0.138	0.145
Isobutyric acid	0.114^b^	0.090^b^	0.167^a^	0.009	0.005
Butyric acid	1.15	1.62	1.47	0.097	0160
Isopentoic acid	0.218^ab^	0.136^b^	0.280^a^	0.017	0.010
Valeric acid	0.286	0.188	0.245	0.017	0.086
Total SCFAs	6.13	6.48	6.11	0.337	0.883

*^1^SCFAs, short-chain fatty acids.*

*^2^Treatments consisted of (1) CON; basal diet, (2) LBPs; basal diet + 4,000 mg/kg LBPs and (3) ABO; basal diet + 20 mg/kg flavomycin + 50 mg/kg quinocetone.*

*^3^SEM, pooled standard error of mean (n = 8).*

*^a,b^Means within each row with different superscripts differ significantly (P < 0.05).*

## Discussion

Weaning stress causes intestinal and immune system dysfunction and reduces pig growth and health (Campbell et al., 2013). Numerous studies have reported that plant-derived polysaccharides (e.g., *Achyranthes bidentata* and *Ganoderma lucidum* polysaccharides) improve immune responses, maintain intestinal structure integrity, balance intestinal microbiota, and reduce diarrhea, which promote pig growth (Li et al., 2015; Hou et al., 2021). In this study, dietary supplementation with either LBPs or ABO increased ADG and decreased the F/G, which may have been attributed to immune response stimulation by LBPs (Zhu et al., 2020). Tan et al. (2019) reported that LBPs, when added to hybrid grouper (*Epinephelus lanceolatus* ♂ × *E. fuscoguttatus* ♀) diets, inhibited hepatic inflammatory responses, increased antioxidant enzyme activity, and improved growth performance and feed efficiency. The intestine has crucial roles in nutrient absorption and defenses against external pathogens (Halas et al., 2010). Wang et al. (2019) reported that dietary LBPs improved intestinal morphology and nutrient absorption in young rats. In addition, Hsieh et al. (2021) indicated that dietary LBPs improved gastric microbiota by increasing gastric *Bifdobacterium* levels in rats. Therefore, these and our evidence may be mediated by the promotional effects of LBPs on growth performance.

Diarrhea incidence has been used as an index to reflect gut health, with a lower diarrheal incidence beneficial for gut health (Pierce et al., 2005). Qiao et al. (2013) reported that diarrheal incidence in piglets was decreased by supplementing medicinal *Aloe vera* polysaccharides. In our study, dietary LBPs or ABO supplementation reduced diarrheal incidence in weaned piglets. Nagy et al. (1992) reported piglet diarrhea after weaning is related to some pathogen levels in the intestine. We previously demonstrated that weaned piglets fed 4,000 mg/kg LBPs had a decreased relative abundance of *Escherichia coli* and *Firmicutes* in the ileum and cecum (Chen et al., 2020). Also, intestinal pH is associated with the proliferation of probiotic microbes, preventing post weaning diarrhea, and maintaining gut enzyme activity (Beuria et al., 2005; Guggenbuhl et al., 2007). Furthermore, Xia et al. (2020) found that dietary LBPs supplementation increased the abundance of *Roseburia faecis, Prevotella* spp., *Butyricicoccus pullicaecorum*, and *Eubacterium uniforme* in mice, which generated particular SCFAs. Thus, LBPs appear to reduce diarrhea incidence in weaned piglets by modulating gut microbiota composition.

Immunoglobulins reflect the immune status of the animal (Yuan et al., 2015; Wang Y. et al., 2018). Hao et al. (2015) reported that the major serum Igs, IgA, IgG, and IgM, were key humoral immunity components in all mammals; they enhance monocyte macrophage phagocytosis and inhibit pathogenic virus and microorganism reproduction (Heidebrecht and Kulozik, 2019; Planchais and Mouquet, 2020). In our study, dietary LBPs supplementation increased serum IgG and IgM levels in weaned piglets. Similarly, Long et al. (2020) reported that broilers fed 2,000 mg/kg LBPs increased serum IgA and IgG levels. Furthermore, the immunoenhancing effects of LBPs may stimulate IL-2 and TNF-α gene expression in human monocytes (Lu and Zhang, 2002). In our study, LBPs dietary supplementation enhanced serum IL-2, IL-10, and TNF-α production in agreement with Ding et al. (2019a), who reported that LBPs administration increased IL-2, IL-6, IL-1, TNF-α, and interferon-γ levels in mice. Littringer et al. (2018) reported that IL-2 and TNF-α were secreted mainly through T helper cells. Wang et al. (2021) found that polysaccharides from traditional Chinese medicines, such as *Artemisia rupestris L., Astragalus, L. barbarum*, and *G. lucidum* regulated immune cell functions and metabolism by activating macrophages and T/B lymphocyte signal pathways. Thus, dietary LBPs appeared to improve the health status of piglets by activating the immune system.

Weaning decreases antioxidation capacity by increasing free radical levels and disrupting oxidative balance (Burke et al., 2009; Yin et al., 2014). Antioxidant parameters such as SOD, GSH-Px, T-AOC, and MDA are routinely used to evaluate antioxidation properties (Hao et al., 2015). SOD degrades superoxide radicals and thus functions as an antioxidant (Urso and Clarkson, 2003). The reduction reaction of lipid peroxides is catalyzed by GSH-Px, and total antioxidative capacity is reflected by T-AOC levels (Aleryani et al., 1998; Tao et al., 2006). MDA is an indicator of lipid peroxidation and reflects the severity of free radical attack on cells (Jiang et al., 2016). It was reported that plant polysaccharides could alleviate this oxidative stress (Liu et al., 2018; Chen and Huang, 2019). In our study, LBPs dietary supplementation increased T-AOC and GSH-Px levels but decreased MDA production. Yang F. et al. (2020) reported that LBPs dietary supplementation relieved oxidative stress in high fat diet-induced obese mice. Liu et al. (2021b) found that LBPs supplementation reduced myocardial oxidative stress via activation of the nuclear factor erythroid-2 antioxidant signal pathway. Furthermore, plants containing flavonoids, phenolic compounds, ascorbic acid, and tocopherol were shown to exhibit antioxidant effects (Wang et al., 2008). It is documented that LBPs were rich in these abovementioned agents (Peng and Tian, 2001). Therefore, we speculated that the antioxidant effects of LBPs may be associated with these components, however, more research is required in this area.

A healthy mucosal structure is key for digestion, physiological function, and growth (Pluske et al., 1996). After weaning, significant changes occur in villus height, crypt depth, and V/C ratios (Cheng et al., 2017). A large V/C ratio represents a greater absorptive efficiency in the small intestine for nutrients, and increased resistance toward disease (Pu et al., 2018). Wang et al. (2019) reported that compound polysaccharide supplementation increased villus height and V/C ratios in the duodenum of young rats. In our study, LBPs dietary supplementation increased villus height and V/C ratios in the duodenum of weaned piglets. Thus, LBPs improved intestinal morphology, maintained intestinal integrity, and promoted intestinal absorption.

A balanced intestinal microbiota is critical for good gut health and nutrition. We observed that some changes had occurred in intestinal microbial composition and metabolism of cecal digesta across groups. PCA revealed that microbial composition and structures were distinct between the CON and LBPs groups, but no differences were determined between the ABO and LBPs groups. Some polysaccharides selectively stimulate the growth and metabolic activity of particular intestinal bacteria associated with health and well-being (Wang et al., 2019). We previously showed that LBPs dietary supplementation decreased the relative abundance of *E. coli* and *Firmicutes* in the ileum and cecum of pigs (Chen et al., 2020). Similar observations by Zhu et al. (2015) showed that polymannuronate addition to broiler diets increased lactic acid bacteria and decreased cecal *E. coli* levels. Furthermore, increased *E. coli* levels may be associated with an increased rate of diarrhea (Zhao et al., 2015). Interestingly, in our study, LBPs dietary supplementation reduced the relative abundance of *Escherichia-Shigella*, *Enterococcaceae*, and *Enterobacteriaceae* in the cecum, and also decreased the diarrhea ratio index. A previous study demonstrated that *Lactobacillus* could protect the intestine by producing antimicrobial agents that suppressed pathogen colonization (Yu et al., 2018). In the current study, dietary supplemental LBPs promoted *Faecalibacterium* and *Lactobacillus* levels. Similarly, Zhao et al. (2015) reported that *mulberry leaf* polysaccharide dietary supplementation reduced the relative abundance of *E. coli* and promoted *Lactobacilli* and *Bifidobacteria* abundance in weaned piglets. Furthermore, Zhu et al. (2020) also reported that LBPs dietary supplementation increased Proteobacteria and Firmicutes abundance, while reducing Bacteroidetes ratios in mice. These results indicated that LBPs could modulate gut microbiota composition and maintain the health of intestinal communities, which may underlie increased growth performance in animal models.

The intestinal digesta contains considerable microbial metabolites and fermentation products that reflect microbial activity and intestinal health (Diao et al., 2014). SCFAs are key metabolites that gut microbiota use to limit inflammation and maintain intestinal integrity to promote gut health (Liu et al., 2020). Thorburn et al. (2015) showed that acetic acid inhibited the histone deacetylase HDAC9.39 to promote regulatory T cell differentiation, with propionic acid enhancing the generation of macrophage. Cresci et al. (2010) reported that butyric acid promoted gut immune responses and preserved intestinal barrier integrity. In addition, intestinal pH was associated with the proliferation of probiotic microbes, the prevention of post weaning diarrhea, and the maintenance of gut enzyme activity (Beuria et al., 2005; Guggenbuhl et al., 2007). Furthermore, LBPs increased SCFAs production which reduced gut environment pH and inhibited *E. coli* levels *in vivo* and *in vitro* studies (Knudsen et al., 2012; Ding et al., 2019b). In our study, LBPs enhanced acetic, propionic, and butyric acid production, and total SCFAs in the cecum, and also promoted *Faecalibacterium* which produced butyrate and generated anti-inflammatory properties (Wan et al., 2019). Therefore, LBPs may have active roles in host immunity and health by modulating gut microbiota and promoting SCFAs production.

## Conclusion

The present research demonstrated that dietary LBPs supplementation improved growth performance, antioxidant capacity and immunity, and reduced diarrhea incidence in weaned piglets. These LBPs effects were associated with a regulatory input on intestinal microbial composition, microbial metabolite production, and intestinal morphology integrity. Thus, LBPs may be used as efficient antibiotic alternatives in weaned piglet feed.

## Data Availability Statement

The datasets presented in this study can be found in online repositories. The names of the repository/repositories and accession number(s) can be found in the article/Supplementary Material.

## Ethics Statement

The animal study was reviewed and approved by the Committee of Animal Care at Hunan Agricultural University (Changsha, China) (permit number: CACAHU 2020-00156).

## Author Contributions

YXY: conceptualization, formal analysis, and writing—original draft. JC: methodology, validation, and resources. FW: data curation. MY: software. BT: funding acquisition. YLY: investigation and supervision. ZY: methodology, project administration, and writing—review and editing. All authors have read and approved the final manuscript.

## Conflict of Interest

The authors declare that the research was conducted in the absence of any commercial or financial relationships that could be construed as a potential conflict of interest.

## Publisher’s Note

All claims expressed in this article are solely those of the authors and do not necessarily represent those of their affiliated organizations, or those of the publisher, the editors and the reviewers. Any product that may be evaluated in this article, or claim that may be made by its manufacturer, is not guaranteed or endorsed by the publisher.
