# Supplementary material for: Lycium barbarum Polysaccharides as Antibiotic Substitutes Improve Growth Performance, Serum Immunity, Antioxidant Status, and Intestinal Health for Weaned Piglets
Source: Front Microbiol. 2022 Feb 25;12:819993. doi: 10.3389/fmicb.2021.819993 (PMC8914510; doi:10.3389/fmicb.2021.819993)
Supplement: Supplementary file 1 [file Data_Sheet_1.PDF]

Supplementary Information

Supplementary table S1. Raw reads and selected effective sequences in each group.

SampleName	Raw PE(#)	Raw Tags(#)	CleanTags(#)	Effective Tags(#)	AvgLen(nt)	Taxon_Tag	OTUs
NP1	67,153	55,811	37,257	34,626	412	31419	607
NP2	68,137	59,236	39,403	37,781	416	35268	580
NP3	64,156	55,752	35,002	32,694	415	29912	655
NP4	63,101	56,062	36,631	34,872	414	33013	585
NP5	68,658	59,725	38,476	36,945	416	33765	641
LP1	74,663	64,716	45,787	44,785	412	43308	592
LP2	71,283	57,286	35,240	34,233	419	31675	634
LP3	73,651	61,828	40,435	39,514	414	37060	668
LP4	67,718	57,163	37,013	35,946	417	34752	430
LP5	72,689	58,262	39,255	38,027	413	36065	554
ALP1	74,005	65,185	46,182	43,014	413	38114	583
ALP2	71,465	63,369	42,723	38,014	414	33612	546
ALP3	76,486	67,685	48,061	45,537	411	42390	589
ALP4	69,260	60,498	40,765	38,408	415	35096	573
ALP5	72,585	63,767	44,355	41,375	413	38223	631

Supplementary table S2. Number of observed species, richness and diversity indices in the caecal sampled from each dietary treatment.

SampleName	observed_species	shannon	simpson	chao1	ACE	goods_coverage
NP1	560	6.71	0.971	576.8	584.638	0.998
NP2	526	6.086	0.959	558.043	568.316	0.998
NP3	655	6.876	0.978	736.743	764.033	0.996
NP4	539	6.499	0.973	577.789	576.799	0.998
NP5	591	6.662	0.97	640.091	640.053	0.997
LP1	521	5.111	0.859	561.513	572.025	0.997
LP2	589	6.372	0.957	617.493	622.73	0.998
LP3	634	7.085	0.982	669.185	679.363	0.997
LP4	379	5.522	0.954	414	412.989	0.998
LP5	510	5.93	0.95	601.2	583.544	0.997
ALP1	539	6.007	0.954	590.776	589.116	0.997
ALP2	514	6.003	0.952	546.515	556.155	0.998
ALP3	532	4.85	0.824	590.679	609.493	0.997
ALP4	532	6.316	0.965	566.169	564.718	0.998
ALP5	564	6.158	0.947	596.015	600.999	0.998

Supplementary Methods

DNA Extraction and PCR Amplification.

Total genomic DNA was isolated from the samples of cecal digesta by using QIAamp DNA Stool Mini Kits according to the manufacturer's instructions. The concentration of the extracted DNA was determined with the NanoDrop-1000 Spectrophotometer (NanoDrop Technologies Inc., Wilmington, DE, USA), DNA purity was monitored on 1% agarose gels. According to the concentration, DNA was diluted to 1ng/μL using sterile water, and was stored at -80℃ before further analysis.

Sequencing was performed at Novogene Bioinformatics Technology Co. Ltd., Beijing, China. The PCR amplifications were conducted with the barcoded primer pair 341f/806r set, which amplifies the V3–V4 fragments of the 16S rDNA gene (341F:CCTAYGGGRBGCASCAG, 806R: GGACTACNNGGGTATCTAAT)

(Muyzer et al., 2013; Caporaso et al., 2011). PCR reactions were performed in a volume of 30 μL containing 12 μL sterile water, 1.0 μL DNA template, 1.0 μL of each primer, and 15 μL 2× Phusion Master Mix (New England Biolabs, USA). The PCR cycle conditions were as follows: initial denaturation at 98 ℃ for 1 min, followed by 30 cycles at 98 ℃ for 10 s, 50 ℃ for 30 s, and 72 ℃ for 30 s, and a final extension step at 72 ℃ for 5 min. Resulting amplicons were confirmed on 2% agarose gels containing ethidium bromide..

All amplicons were in the size range of 400–450 bp, and were purified using a GeneJET Gel Extraction Kit (Thermo Fisher Scientific, Carlsbad, CA, USA). Following quantitation, equal concentrations of the purified amplicons were combined into a single tube. Sequencing libraries were generated using a NEB Next Ultra DNA Library Prep Kit for Illumina (New England Biolabs, Ipswich, MA, USA) following manufacturer's recommendations, and index codes were added. The library quality was assessed on a Qubit @ 2.0 Fluorometer (Thermo Fisher Scientific, Carlsbad, CA, USA) and Agilent Bioanalyzer 2100 system. At last, the library was sequenced on an IlluminaHiSeq2500 platform and 250 bp paired-end.

reads were generated.**Bioinformatics analysis**

Paired-end reads was assigned to samples based on their unique barcode and truncated by cutting off the barcode and primer sequence. Paired-end reads were merged using FLASH (V1.2.7,http://ccb.jhu.edu/software/FLASH/) [1],a very fast and accurate analysis tool, which was designed to merge paired-end reads when at least some of the reads overlap the read generated from the opposite end of the same DNA fragment, and the splicing sequences were called raw tags. The tags were compared with the reference database (Gold database, http://drive5.com/uchime/uchime_download.html)using UCHIME algorithm (UCHIME Algorithm, http://www.drive5.com/usearch/manual/uchime_algo.html) [4] to detect chimera sequences, and then the chimera sequences were removed [5]. Then the Effective Tags finally obtained. Sequences analysis were performed by Uparse software (Uparse v7.0.1001， http://drive5.com/uparse/) [6]. Sequences with ≥97% similarity were assigned to the same OTUs. Representative sequence for each OTU was screened for

further annotation. For each representative sequence, the GreenGene Database 3(http://greengenes.lbl.gov/cgi-bin/nph-index.cgi) [7] was used based on RDP classifier(Version 2.2, http://sourceforge.net/projects/rdp-classifier/)[8]algorithmto annotate taxonomic information.

In order to study phylogenetic relationship of different OTUs, and the difference of the dominant species in different samples(groups), multiple sequence alignment were conducted using the MUSCLE software (Version 3.8.31 , http://www.drive5.com/muscle/)[9]. OTUs abundance information were normalized using a standard of sequence number corresponding to the sample with the least sequences.

Alpha diversity is applied in analyzing complexity of species diversity for a sample through 6 indices, including Observed-species, Chao1, Shannon, Simpson, ACE, Good-coverage. All this indices in our samples were calculated with QIIME (Version 1.7.0) and displayed with R software (Version 2.15.3). Beta diversity analysis was used to evaluate differences of samples in species complexity, Beta diversity on both weighted and unweighted unifrac were calculated by QIIME software (Version 1.7.0). Unweighted Pair-group Method with Arithmetic Means (UPGMA) Clustering was performed as a type of hierarchical clustering method to interpret the distance matrix using average linkage and was conducted by QIIME software (Version 1.7.0).

Reference

[1] Magoč T, Salzberg S L. FLASH: fast length adjustment of short reads to improve genome assemblies. Bioinformatics 27.21 (2011): 2957-2963.

[2] Bokulich, Nicholas A., et al. Quality-filtering vastly improves diversity estimates from Illuminaamplicon sequencing. Nature methods 10.1 (2013): 57-59.

[3] Caporaso, J. Gregory, et al. QIIME allows analysis of high-throughput community sequencing data. Nature methods 7.5 (2010): 335-336.

[4] Edgar, Robert C., et al. UCHIME improves sensitivity and speed of chimera detection. Bioinformatics 27.16 (2011): 2194-2200.

[5] Haas, Brian J., et al. Chimeric 16S rRNA sequence formation and detection in Sanger and 454-pyrosequenced PCR amplicons.Genome research 21.3 (2011):

494-504.
[6] Edgar, Robert C. UPARSE: highly accurate OTU sequences from microbial amplicon reads. Nature methods 10.10 (2013): 996-998.
[7] DeSantis, Todd Z., et al. Greengenes, a chimera-checked 16S rRNA gene database and workbench compatible with ARB. Applied and environmental microbiology 72.7 (2006): 5069-5072.
[8] Wang, Qiong, et al. Naive Bayesian classifier for rapid assignment of rRNA sequences into the new bacterial taxonomy. Applied and environmental microbiology 73.16 (2007): 5261-5267.
[9] Edgar R C. MUSCLE: multiple sequence alignment with high accuracy and high throughput. Nucleic acids research32.5 (2004): 1792-1797.
