# Supplementary material for: Lycium barbarum Polysaccharides as Antibiotic Substitutes Improve Growth Performance, Serum Immunity, Antioxidant Status, and Intestinal Health for Weaned Piglets
Source: Front Microbiol. 2022 Feb 25;12:819993. doi: 10.3389/fmicb.2021.819993 (PMC8914510; doi:10.3389/fmicb.2021.819993)
Supplement: Supplementary file 2 [file Data_Sheet_2.docx]

**Supplementary data**


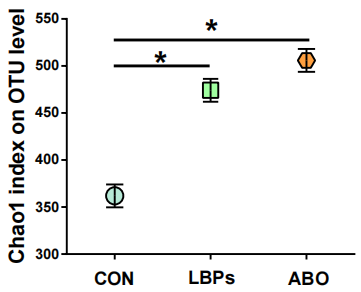

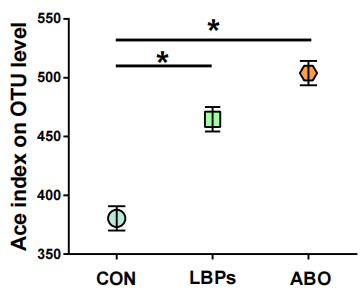

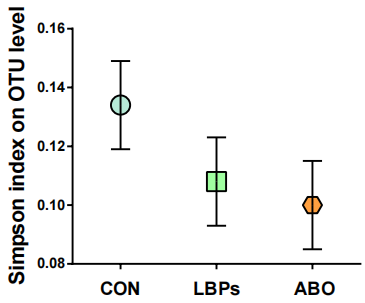

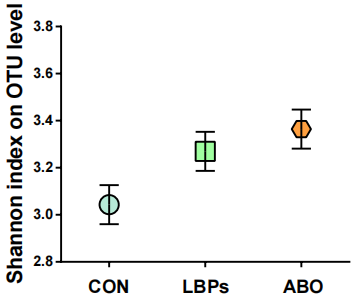

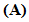

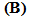

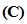

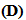


**Fig S1.** The richiness and divsity index of species at 97% similarity level: Chao index (A), Ace index (B), Shannon index (C) and Simpson index (D). CON: basal diet; LBPs: basal diet + 4000mg/kg LBPs ; ABO: basal diet + 20 mg/kg flavomycin +50 mg/kg quinocetone. Asterisks indicate statistical differences between different groups: * *P* < 0.05.


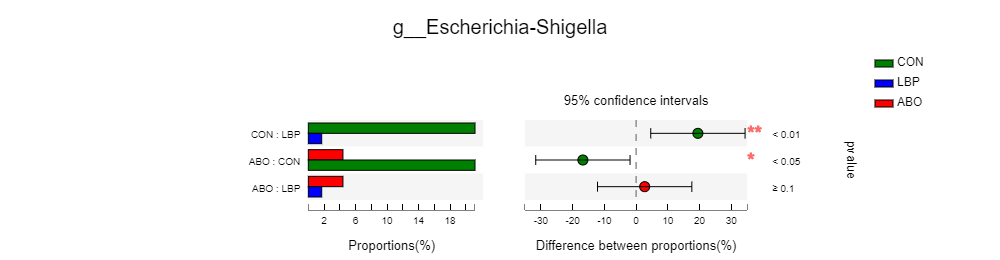

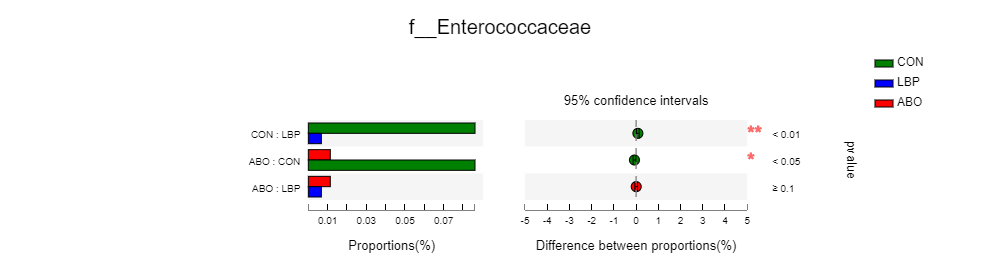


**Fig S2.** Comparative analysis of 3 most relative abundances of gut microbiota. Kruskal–Wallis test followed by Tukey test was used to evaluate the statistical significance. Asterisks express statistical differences between different groups: *0.01 < *P* ≤ 0.05, **0.001 < *P* ≤ 0.01, *** *P* ≤ 0.001. CON: basal diet; LBP: basal diet + 4000mg/kg LBPs; ABO: basal diet + 20 mg/kg flavomycin +50 mg/kg quinocetone.
